# Risk Assessment of Heavy Metals in Road-Deposited Sediments and Correlation Distribution of DOM and Heavy Metals in Beijing, China

**DOI:** 10.3390/toxics13040308

**Published:** 2025-04-16

**Authors:** Donghai Yuan, Pengmiao Li, Chenling Yan, Jinggang Wang, Xiaochen Bai, Yuhang Wei, Chen Wang, Yingying Kou

**Affiliations:** 1 Key Laboratory of Urban Stormwater System and Water Environment, Ministry of Education, Beijing University of Civil Engineering and Architecture, Beijing 100044, China; yuandonghai@bucea.edu.cn (D.Y.); lpm13935873154@163.com (P.L.); 13051902242@163.com (X.B.); sdtcwyh@163.com (Y.W.); 2 Beijing Key Laboratory of Municipal Solid Waste Detection Analysis and Evaluation, Beijing Municipal Institute of City Management, Beijing 100028, China; yanchenling@aliyun.com; 3 School of Information Technology, Nanchang Vocational University, Nanchang 330007, China; jingg-wang@163.com; 4 China Academy of Urban Planning and Design (Beijing) Planning & Design Consultants Co., Ltd., Beijing 100044, China; sdjnkyy@163.com

**Keywords:** heavy metals, dissolved organic matter, road sediments, risk assessment, correlation

## Abstract

Road-deposited sediments (RDS) from 28 sites in Beijing were studied and analyzed for eight heavy metals. In RDS, the levels of Cr, Ni, Cu, Zn, As, Cd, Pb and V were 2.76, 1.11, 2.40, 1.65, 1.09, 6.52, 4.13 and 0.06 times the background values, respectively. The levels were rated in accordance with the geoaccumulation index (Igeo) as follows: Cd>Pb>Zn>Cu>Cr>Ni>As>V. In the four functional zones, the potential ecological risk index method showed that most of the heavy metals have environmental risk index values ($E_{r}^{i}$) of less than 40, but the multifactor environmental risk value (RI) for Pb in the transportation area exceeded 150. Four functional areas had Cd values greater than 160, exhibiting the highest risk. The human health risk assessment revealed that exposure pathways followed this decreasing order: ingestion > dermal > inhalation. Three DOM fractions were resolved in the sediments of the four functional zones, including terrestrial fulvic-acid-like fractions (C1), humic-acid-like fractions (C2), and tryptophan-like fractions (C3), and the DOM fractions were affected by both exogenous and endogenous sources. A positive correlation existed between DOM and Ni and Cu in the transportation zone, and the correlation between DOM and heavy metals in other zones was not apparent. In conclusion, heavy metals in different functional zones affected the concentration and characteristics of DOM, and there was a strong correlation between heavy metals and DOM concentration and features.

## 1. Introduction

High urban population densities lead to the consumption of large amounts of resources and the generation of large quantities of waste, and human activities pose numerous hazards to the environment [1]. The physical behavior and chemical components of road-deposited sediment (RDS) are influenced by deposition, redistribution, transport, and chemical reactions from multiple sources [2]. Recent studies have extensively demonstrated that RDS, as a primary carrier of heavy metals and other pollutants, serves as a significant source of urban runoff pollution [3,4].

Heavy metals have received widespread attention due to their complex degradation, which is highly toxic and insidious [5]. Particularly in urban areas, heavy metals serve as primary contaminants [6]. Heavy metals in RDS can migrate into water bodies through surface runoff by scouring and infiltration processes [7], posing significant risks to aquatic ecosystems [8]. Atmospheric dust deposition, vehicle abrasives, petrol combustion, road paints, etc. are the primary contributors of heavy metals within RDS [9]. Heavy metals such as Cd, Cu, and Pb are found in the pigments used for road signs [10]. Ordóñez et al. showed that mechanical wear and leakage of lubricants from vehicle tires and bodies and wear of anticorrosive galvanized automotive panels cause increased Zn levels in road sediments [11]. Human health can also be negatively impacted by heavy metal exposure and the integration of heavy metals into the food chain [12]. Consideration of the severity of heavy metal contamination in RDS in large cities is necessary for the protection of the environment and the people [13].

DOM is a class of mixtures of various organic compounds containing carbonyl, methoxy, conjugated double bond, carboxyl, hydroxyl, and other functional groups. Because of their fluorescence properties, hydrophilicity, molecular weight, and biodegradability, it can be further classified into different subunits, including lignin, humic acid, protein compounds, lipids, carbohydrates, organic acids, etc. [14]. DOM in RDS is commonly influenced by soil erosion and hydrological runoff while readily interacting with heavy metals in the environment. On the one hand, heavy metals can directly or indirectly affect DOM characteristics in terrestrial and aquatic environments [15]. On the other hand, DOM can be enriched by adsorption complexation, ion exchange, etc., as well as by a series of reactions with heavy metals and nutrient elements, thus affecting their environmental migration process [16]. Analyzing the compositional distribution characteristics of DOM in road-deposited sediments (RDS) can help elucidate the migration and enrichment mechanisms of heavy metals.

This study systematically investigates the interaction mechanisms between heavy metals and DOM in RDS across representative functional zones of Beijing. Although previous studies have examined the basic characteristics of heavy metals and DOM in urban environments, there remains a lack of in-depth understanding regarding their spatial distribution patterns in RDS across different functional zones of megacities. The present study focuses on the following aspects: (1) elucidating the spatial distribution patterns of heavy metals and DOM components across different functional zones; (2) analyzing the relationship between DOM characteristics and heavy metals; (3) conducting ecological risk assessments of RDS in different functional zones. This study reveals the pollution characteristics of RDS under the complex environmental conditions of the metropolis and provide a theoretical basis for its ecological risk control; at the same time, through the evaluation of ecological risk of heavy metals, we can accurately identify the degree of contamination and spatial distribution characteristics so as to guide the management department to lock high-risk areas and realize the optimal allocation of management resources.

## 2. Materials and Methods

### 2.1. Study Area

Beijing is China’s political, cultural, economic, and transportation center, with a rich historical and cultural heritage, modern urban facilities, and high levels of development. The main pattern of downtown Beijing is horizontal and vertical, with the five ring roads encircling the city in layers, constituting the overall urban pattern of Beijing, and higher population density within the fifth ring road [17]. Beijing has a large population, with a resident population of 21.843 million at the end of 2022 according to the Beijing Municipal Bureau of Statistics, consuming enormous resources and simultaneously creating and emitting large amounts of pollutants.

### 2.2. Sample Collection and Analysis

**Figure 1:**
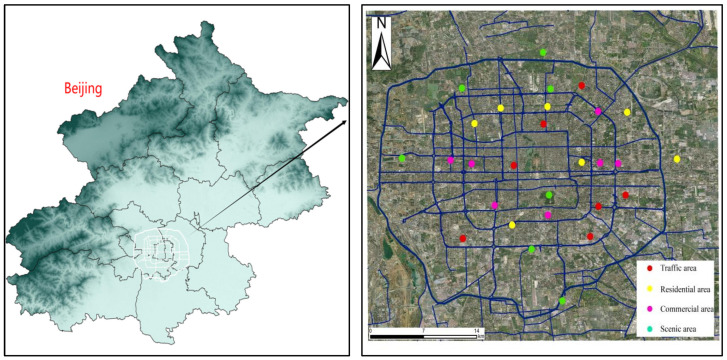
Locations in the study area. Red: traffic area; yellow: residential area; pink: commercial area; green: Scenic area.

This study adopted a network-distribution method to establish sampling points in Beijing’s downtown area, considering functional zone types and the ring-road divisions. Specific sampling strategies included: traffic areas—selecting major roads with high average daily traffic flow; residential areas—choosing high-density residential zones, ensuring no industrial pollution sources existed within a 3-km radius; commercial areas—focusing on large-scale commercial complexes; scenic areas—targeting zones designated for landscape protection or recreational purposes. At least five sampling points were set for each functional area, covering the second to fifth ring roads. The methodology referenced the Beijing Urban Design Guidelines and U.S. EPA standards for soil and sediment data to ensure quality control. The sampling network’s spatial configuration is displayed in Figure 1. In 2022, sampling was conducted in the study area at 28 selected sampling points under clear weather conditions, with wind speeds below 1 m/s and no rainfall in the preceding three days. Quality control was performed by simultaneously collecting field blank samples and parallel samples (HJ/T 166-2004) [18]. Road-deposited sediments were collected using a plastic brush and dustpans. Samples from each site were immediately sealed in zip-lock polyethylene bags and transported to the laboratory under refrigeration (4 °C) within 24 h to minimize contamination and degradation [19,20]. Samples are air-dried and sieved to remove debris [21].

Heavy metal leaching was performed using the electrothermal plate ablation method, with the following steps: 0.5 g road sediment sample was weighed into a 50 mL polytetrafluoroethylene crucible, followed by sequential addition of 10 mL nitric acid, 5 mL hydrofluoric acid, and 5 mL perchloric acid. The mixture was heated on an electro-thermal plate at 120–150 °C for approximately 1 h with the lid covered. Subsequently, the lid was removed, and heating continued to drive off silicon until white perchloric acid fumes appeared. The lid was then replaced to facilitate organic carbon decomposition. After the black organic residues on the inner crucible walls were completely dissolved, the lid was opened to disperse the fumes, and heating continued until a viscous residue remained. The resulting solution was analyzed by inductively coupled plasma mass spectrometry (ICP-MS, model: NexION 300; manufacturer: PerkinElmer, Inc. (Waltham, MA, USA)). Calibration curves were established with matrix-matched multielement standards (R^2^ > 0.999). Background contamination was controlled through reagent blanks (synchronized acid processing blanks with target element concentrations below the method detection limit) and instrumental blanks (periodic runs of ultrapure water or diluted acid). Quality control included 10% sample replicates (RSD < 5%) and at least one parallel sample analysis to ensure data reliability and procedural consistency [22]. Eight representative heavy metals, Cu, As, Mn, Ni, Cd, Cr, Zn, Pb, and V, were selected [23]. Analysis of heavy metals in each sample was repeated three times during the experiment, and strict quality control was performed. The water–soil ratio was 10:1 [24,25,26]. The DOM sample was obtained by oscillating centrifugation operation, taking the supernatant and passing it through a 0.45 μm membrane. Following filtration, DOM samples were stored in a 4 °C refrigerator and measured as soon as possible. All heavy metal concentrations in this study were determined based on dry weight (dw).

### 2.3. Heavy Metal Evaluation Methods

This study employed three evaluation methods: the geoaccumulation index (Igeo), used to quantitatively assess the degree of heavy metal contamination in sediment [27,28,29,30]; the potential ecological risk index (RI) proposed by Hakanson, applied to evaluate the combined ecological hazards of multiple heavy metals [31,32,33,34,35]; and the health risk assessment method based on U.S. EPA guidelines, which involves calculating the hazard quotient (HQ), hazard index (HI), and carcinogenic risk (CR) through ingestion, inhalation, and dermal contact pathways to assess noncarcinogenic effects and carcinogenic risks [36,37]. The risk calculation formulas are presented below (Formulas (1)–(11)). Detailed parameters and their explanations can be found in Table S1 and the formula section of the Supplementary Materials [38,39,40,41,42].

(1) $$\begin{array}{c} I_{geo}=\log_{2}\left[\frac{C_{i}}{k\times B_{i}}\right] \end{array}$$

(2) $$\begin{array}{c} C_{f}^{i}=C_{s}^{i}/C_{n}^{i} \end{array}$$

(3) $$\begin{array}{c} E_{r}^{i}=T_{r}^{i}\times C_{f}^{i} \end{array}$$

(4) $$\begin{array}{c} RI=\sum_{i=1}^{n}E_{r}^{i}=\sum_{i=1}^{n}T_{r}^{i}\times C_{f}^{i}=\sum_{i=1}^{n}T_{r}^{i}\times \frac{C_{s}^{i}}{C_{n}^{i}} \end{array}$$

(5) $$CDI_{ing}=\frac{CS\times EF\times ED\times IRS}{BW\times AT\times 10^{6}}$$

(6) $$CDI_{inh}=\frac{PM\times CS\times ET\times EF\times IR_{air}\times ED}{BW\times PEF\times AT}$$

(7) $$CDI_{dermal}=\frac{CS\times SA\times AF\times EF\times ED\times ABS}{BW\times AT\times 10^{6}}$$

(8) $$HQ=\frac{{CDI}_{i}}{R_{f}D}$$

(9) $$HI=\sum_{i=1}^{n}HQ_{k}=HQ_{inh}+HQ_{ing}+HQ_{dermal}$$

(10) $$CR_{i}={CSF}_{i}\times {CDI}_{i}$$

(11) $$CR=\sum_{i=1}^{n}{CR}_{i}$$

### 2.4. DOM Analysis

Fluorescence composition of DOM was determined by parallel factor analysis (PARAFAC) using the DOMFlour module within the MATLAB 2021 software [43]. Each component’s concentration was expressed according to its highest fluorescence intensity (F_max_) [44]. To accurately analyze the connection between DOM components and indices, principal component analysis was used. As measures of the origin and nature of DOM, fluorescence index (FI), biogenic index (BIX), humification index (HIX), and β:α values were used. SUVA_254_ and E4/E6 reflected the degree to which organic matter became humified and aromatized, SUVA_260_ demonstrated the amount of hydrophobic components in the organic matter, A_250_/A_365_ reflected the molecular weight of the organic matter, A_253_/A_203_ characterized the variety of substituents on the aromatic ring structure in DOM, and A_240-400_ characterized the changes in the aromatic compounds in the DOM.

### 2.5. Statistical Analysis

The preprocessed 3D fluorescence data were analyzed in MATLAB. SPSS Statistics 27 was used for data processing and analyzing the association between contaminants and DOM using Pearson correlation. The sample point distribution was mapped using ArcGIS 10.8.2, and additional images were completed using Origin 2021.

## 3. Results and Discussion

### 3.1. Heavy Metal Content and Pollution Characteristics

The heavy metal content and background values in RDS are shown in Table S2. The levels of Cr, Ni, Cu, Zn, As, Cd, and Pb were 2.76, 1.11, 2.40, 1.65, 1.09, 6.52, and 4.13 times the background values, respectively, showing a certain degree of enrichment, which should be taken seriously. As, Ni, and Cu showed relatively high coefficients of variation, more obviously disturbed by the outside world than other elements.

V in all four functional areas was below the Beijing background value. The background values of Cr, Ni, Cu, Zn, As, Cd, Pb, and V are 29.8, 24.7, 29.8, 18.7, 57.5, 7.7, 0.12, 24.6, and 79.2 mg/kg, respectively [4,45]. In the traffic area, the levels of Cr, Ni, Cu, Zn, As, Cd, and Pb were 2.98, 1.17, 2.90, 2.15, 1.12, 9.32 and 5.27 times the background values, respectively. In residential areas, the levels of Cr, Ni, Cu, Zn, As, Cd, and Pb are 2.47, 0.98, 1.87, 1.48, 0.95, 5.42 and 3.24 times the background value, respectively. In scenic areas, the levels of Cr, Ni, Cu, Zn, As, Cd, and Pb were 2.82, 1.25, 2.00, 1.44, 1.12, 5.64, and 3.56 times the background values, respectively. In commercial areas, the levels of Cr, Ni, Cu, Zn, As, Cd and Pb were 2.60, 0.90, 2.67, 1.52, 1.08, 5.49, and 4.25 times the background value, respectively. Heavy metals in traffic, residential, scenic, and commercial areas showed different degrees of enrichment [45]. The concentration of Cd significantly surpassed that of other heavy metals, which may indicate that anthropogenic activities might significantly impact the levels of contaminants such as heavy metals in RDS [46]. This high concentration may also owe to heavy metals such as Cd, Cu, Pb, and others being present in the tinted paint used for pavement markings [9]. The traffic area is located in a place of high traffic flow in Beijing. The road surface is asphalt and contains colored paints. Beijing is windy all year round, and heavy metals carried by the road’s surroundings can also diffuse and settle to the road because of the climate; anthropogenic and climatic factors, among others, can impact the concentration of heavy metals in RDS. Therefore, in Beijing’s transportation zones, the concentration of heavy metals can be reduced by adopting more environmentally friendly paints and so on.

**Figure 2:**
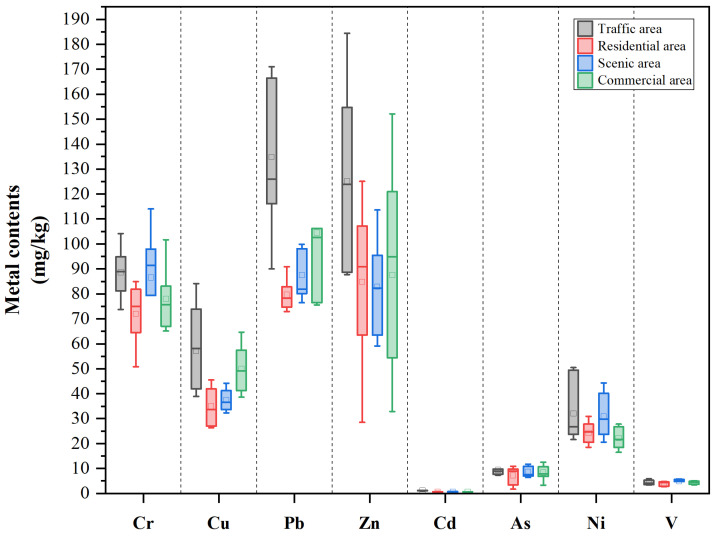
Boxplot of heavy metal concentrations in different functional zones, with gray, red, blue, and green representing data from the traffic area, residential area, scenic area, and commercial area, respectively.

The heavy metal contents are shown in Figure 2 and Figure S1. The content of each heavy metal was the highest in the second ring. The concentrations of Zn and Pb varied across the different ring roads, with average Zn levels of 123.85, 83.64, 98.39, and 86.93 mg/kg in the second to fifth rings, respectively, and Pb levels of 114.08, 96.44, 107.73, and 87.08 mg/kg. These heavy metals likely originated from three primary sources: vehicle wear on highways, street dust resuspension, and gasoline combustion emissions [47]. In contrast, the second ring urban area, as the most central area of the city, not only possesses a long history but has a higher intensity of human activities than the other rings. Hence, the fifth ring suffered from the lowest amount of contamination of these three heavy metals. In addition, the presence of Pb, Zn, and Cu in decorative materials, pipes, batteries, and consumer goods and packaging waste may result in their environmental persistence and chronic pollution. The study revealed consistent distribution patterns of Pb and Cr across different ring roads (second ring > fourth ring > third ring > fifth ring), indicating their potential common pollution sources, which aligns with the findings of Wang et al. [48]. Additionally, Ni contamination was attributed primarily to industrial processes including fossil fuel combustion, fuel oil utilization, and metal smelting.

### 3.2. Ecological Risk Evaluation of Heavy Metals and the Extent of Contamination

According to the Igeo analysis results (Figure S2), heavy metal pollution in Beijing’s RDS ranged from clean to moderate levels, with the pollution degree ranking as follows: Cd>Pb≈Zn≈Cu≈Cr>Ni≈As>V. Igeo values for metal V were negative in all four functional areas, indicating that Beijing’s pavements are not contaminated with V. The Igeo values of As and Ni were partly negative, but the Igeo values were less than 1, indicating mild contamination. Compared with Ni, As, and V, metals such as Cd, Pb, Cr, Cu, and Zn demonstrated significantly higher contamination levels. Notably, within the study area, Cr, Cu, and Zn exhibited Igeo values exceeding 1. This indicates that contamination levels were moderate. In the transportation zone, the Igeo values of the heavy metal Pb ranged from 1.29 to 2.21, indicating moderate to severe pollution, and the degree of Pb pollution of the rest of the functional areas was moderate. Cd’s Igeo scores varied between 1 and 3 in the four functional regions. Still, as shown in Table S3, the Igeo values of the traffic and commercial areas were a bit higher, representing higher levels of contamination in transportation and commercial areas compared with residential and scenic areas. Scenic regions may be affected by factors such as large amounts of vegetation, so they were less polluted than the other functional areas.

From Table S3, in the four major functional areas, $E_{r}^{i}$ values for most heavy metals fell below the threshold of 40, suggesting low ecological risk in these areas. However, the RI value of Pb in the traffic zone was more than 150, which indicated a higher risk to the ecological environment. However, the Pb RI in the other functional zones did not exceed this value, and the risk was lower. Cd’s $E_{r}^{i}$ values exceeded 160 in all four functional areas, indicating the highest risk for Cd. Among the four zones, the transportation district had a higher risk level than the other three districts, followed by the commercial and residential areas, and the scenic area was the least contaminated. Probably because of the large population and high flow of people in Beijing, compared with the other districts, Beijing’s business and residential sectors had greater levels of heavy metal pollution.

The $E_{r}^{i}$ and Igeo of Cd were higher than those of all other metals, possibly because of the presence of Cd metal in diesel fuel, lubricants, and tire wear [49,50]. High ambient temperatures and the abrasion of vehicle tires on the road, among other things, may result in the discharge of Cd into the surroundings. Diesel and lubricant leakage can also lead to the release of Cd. Therefore, congested traffic in Beijing may be responsible for high Cd concentrations [51]. Pigments used for road signs contain elements such as Cd, Cu, and Pb [10]; in particular, the yellow paint used in pavement marking has high content of Cd, an element that typically enters the environment via human activities. Pb and Zn are also known as traffic pollutants because they are found in automobile emissions [52,53]; pollutants from vehicle exhaust can accumulate on road surfaces through ground deposition and adsorption of road dust [54]. Thus, it can be explained why the heavy metal Igeo values in traffic regions were greater than those in the other three functional zones. The environmental sources of the elements Pb and Zn are similar, namely motor vehicle traffic and oxidation of motor vehicle lubricants [55], with factors such as corrosion of metal structures, deteriorating paint, and geological processes contributing significantly to their accumulation. This suggests that Beijing should give more attention to improving the environmental quality of transportation areas in the future.

**Figure 3:**
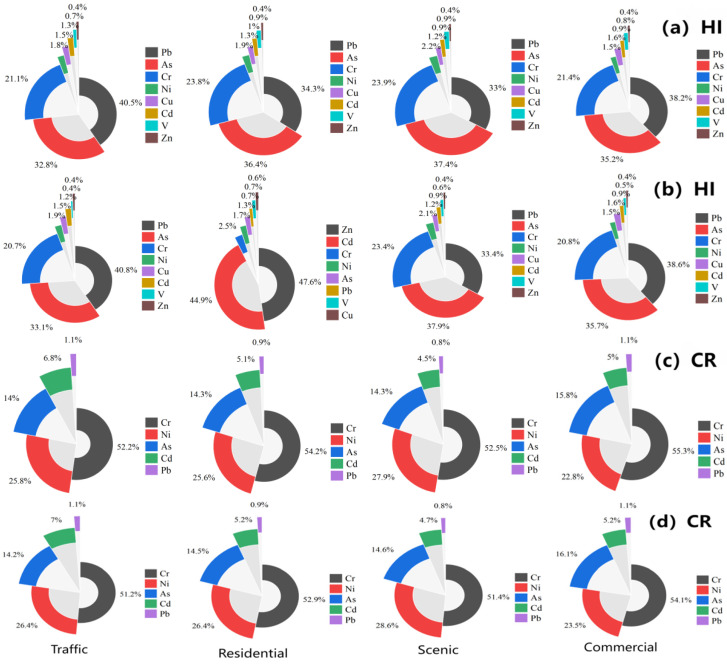
The left to right columns represent traffic, residential, scenic, and commercial areas, respectively; from top to bottom: (**a**) adult noncarcinogenic (HI) contribution ratio; (**b**) minor noncarcinogenic (HI) contribution ratio; (**c**) adult carcinogenic risk (cancer risk, CR) contribution ratio; (**d**) minor carcinogenic risk (CR) contribution ratio.

Figure 3 and Table S4 display the HI and CR values. As shown in the data, the noncarcinogenic danger to the public was virtually nonexistent, as the HI values corresponding to the heavy metals in the four areas were lower than one for both adults and children. Compared with adults, children had a higher risk of hand–oral ingestion and skin contact and a lower risk of inhalation. Other scholars have also reported similar trends [56,57]. For noncarcinogenic risk, oral ingestion was the primary mode of ingestion, and children were more exposed than adults in all three routes. For the same heavy metal, the exposure to heavy metal contamination was greatest under the hand–oral route, followed by dermal contact, and the smallest by respiratory inhalation [58]. It was concluded that children are particularly vulnerable. Their frequent hand-to-mouth activities may lead to a much higher daily dust intake via the hand-to-mouth route than adults; consequently, children are more exposed than adults. Among the four functional zones, the highest noncarcinogenic risk was found for Pb, followed by As and Cr in minors and adults. In minors, these three heavy metals’ HI values were greater than 0.1, meaning that children’s health needs to be protected. Transportation areas had the highest HI values, followed by commercial, scenic, and residential regions. This might be due to human activities such as exhaust emissions in transportation and commercial areas.

For both adults and children, the oral intake and dermal exposure routes contributed more to the CR values. The probability of cancer risk (CR) values ranged between 5.79 × 10^−7^ and 2.16 × 10^−5^ for adults and 9.57 × 10^−6^ and 6.70 × 10^−4^ for children; the highest probability of cancer risk values for both adults and children was found in the transportation zone, followed by the commercial, scenic, and residential areas. Among adults, the CR values in the transportation zone were between the acceptable range of 1 × 10^−6^ and 1 × 10^−4^; the exposure did not cause significant health effects. The small percentage of CR < 10^−6^ showed that there was little chance of heavy metals causing cancer in humans. For children, the carcinogenic risk (CR) values of Cr, Ni, and As exceeded the 1 × 10^−4^ safety threshold across all four functional zones, indicating unacceptable carcinogenic exposure. To prevent the public health risk from RDS, it may be useful to reduce time spent outdoors, wear masks, etc.

### 3.3. Changes in DOM Quantity and Quality

Previous researchers classified the fluorescence peaks of 3D-EEMs into four types [59]. Three fluorescence components, component 1 (C1), component 2 (C2), and component 3 (C3), were identified in the DOM in the Beijing pavement sediment using three-dimensional fluorescence combined with parallel factor analysis (EEM-PARAFAC), as shown in Figure S4. Among them, C1 (Ex/Em = 250/400 nm): terrestrial humic acid [43]; C2 (Ex/Em = 350/450): visible humic-like acid; C3 (Ex/Em = 275 /350): protein-like region [60], mainly represented as a tryptophan-like substance [61].

The structural properties of DOM can be understood through EEM characterization. As can be seen in Figure S3, for all 28 sites, C1, C2, and C3 fractions were detected. The C1–C3 fractions of DOM were influenced by the functional regions, and it can be seen from Figure S4 that C1 was absolutely dominant, followed by C2 and C3. The humic-like (C1 + C2) fractions were generally mainly related to exogenous inputs, and the protein-like fractions (C3) were generally considered as the microbial breakdown of aquatic plant or algae leftovers, etc. From the samples collected from the four functional zones, the C1 peak was clearly visible in the EEM spectra. In particular, the C1 peaks were dominant in traffic and commercial zones, suggesting the presence of a large amount of humus-like material in these two functional zones. A large amount of protein-like material was significantly observed in residential areas (tryptophan, native DOM) [62]. The higher C2 in the landscape area relative to the other three functional areas suggests that the landscape area contains higher humus content, which may be due to the influence of plants such as deciduous leaves in the landscape area.

**Figure 4:**
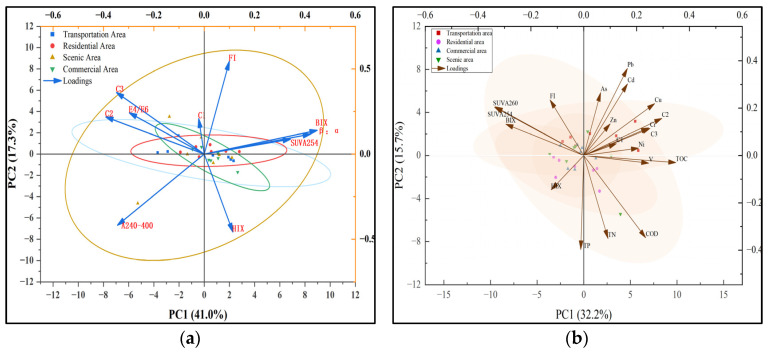
(**a**) Principal component analysis loading plots of components in DOM of different functional areas; (**b**) principal component analysis of heavy metals and DOM fractions in sediments.

The principal component analysis (PCA) factor loadings for each functional area are displayed in Figure 4a. The figure illustrates the variation of samples from various functional areas. Most of the samples were relatively dispersed indicating that the variation in DOM components in RDS varies more with functional areas. The dispersion was most pronounced in the transportation area, which may have been due to the pollution situation as well as the impact of anthropogenic activities being more pronounced in the transportation area than in other functional areas. In this study, Bartlett’s test was performed before principal component analysis, and the resulting value is less than 0.05, which indicates that there was a correlation between the variables and that the data were suitable for factor analysis. Principal components 1 and 2 together explained 58.3% of the variation in these parameters (Figure 4a). Principal component 1 (PC1) explained 41.0% of the variations and showed good positive correlation with β:α, BIX, and SUVA_254_, as wella s negative correlations with C2, C3, A240-400, and E4/E6. Principal component 2 (PC2) explained 17.3% of the total variance and showed better positive correlations with FI, C3, C2, E4/E6, and C1, as well as negative correlations with HIX and A240-400. Among them, the loadings of FI, HIX, A240-400, BIX, β:α, C2, and C3 were larger, indicating that these elements had higher contributions in different functional areas. In addition, the distance of the loadings of each variable can also reflect some information, as closer distance indicates that two variables have some correlation. For example, the loadings of β:α, BIX, and SUVA_254_ were close to each other, which indicates that they had some correlation. Four ellipses were obtained based on the sampling point score matrix, which indicated that DOM features changed with functional areas. The higher scores of the sampling points in the scenic area indicated that the DOM content was highest in the scenic area, followed by the transportation area, residential area, and commercial area, which may have been due to the fact that the DOM in scenic area and the transportation area was deeply affected by human activities.

The degrees of dispersion of the DOM values in the RDS in the traffic, scenic, residential and commercial areas obtained from the study are described in Table S5. Among the different functional zones, the scenic zone’s highest mean values for SUVA_254_, SUVA_260_, A_253/203_, and A_240-400_ all showed that the degree of aromatization and hydrophobicity of DOM in the pavement sediment in scenic zone was the highest, that the degree of benzene ring substitution in the DOM was relatively high, and that the degree of DOM was the most aromatized. SUVA_254_, SUVA_260_, A_253/203_ had the lowest mean values. A_240-400_ had the lowest mean values in the commercial area, indicating that DOM had the lowest degree of aromatization in the commercial area; A_250/A365_ had the highest mean values in the transportation area, indicating that DOM had the lowest molecular weight, and vice versa in the scenic area. The mean values of E4/E6 in the four functional zones were relatively close to each other, indicating that the degree of decay, relative molecular mass, and degree of polymerization of the DOM in the RDS in each functional zone were relatively close to each other as a whole from the E4/E6 point of view.

From Figure S5 and Table S5, the FI value of the traffic area at point S12 was greater than 1.9. The FI values at points S17 and S20 in scenic area were greater than 1.9. In the commercial area, the FI at point S26 exceeded 1.9. This indicates that the humus contained in the DOM in the RDS at these points was mainly of autochthonous origin. The FI values at the points in the remaining functional areas ranged from 1.4–1.9, which indicated that the DOM in the RDS at these points was of mixed origin. The average FI values for the four functional areas were 1.84, 1.86, 1.89, and 1.82, respectively, indicating that the proportion of biogenic sources of DOM in the RDS in the scenic area was high and less aromatic.

From Figure S6 and Table S5, the BIX values of points S12, S16, S23, and S24 in the traffic zone; points S5, S6, and S27 in the residential zone; points S4, S26, and S28 in the scenic area; and points S9, S10, S11, S13, and S20 in the commercial area were higher than 0.8, suggesting that these points’ DOM in RDS had strong autochthonous source characteristics and was produced mainly by endogenous biological activities. All other BIX values were less than 0.8, indicating that the autochthonous source contribution of DOM in the RDS at other points was low. The average HIX values for residential, traffic, commercial, and scenic areas were 1.86, 2.17, 2.20 and 2.61, respectively, which showed a gradual increasing trend. This indicates that the degree of humification of the DOM in the scenic area was higher than that in the other functional areas. The residential area had the highest mean value of β:α among the four major functional zones, which indicated that the biological activity of DOM in the sediment in the residential area was higher in comparison with the other zones.

In summary, the DOM composition was affected by a combination of exogenous inputs and endogenous enrichment, and the humic-acid-like fraction was significantly higher than the protein-like fraction. Therefore, to protect the environment of Beijing pavement sediments, it is necessary to take into account both the role of endogenous release and the control of exogenous anthropogenic inputs.

### 3.4. Correlation Between DOM and Heavy Metals

DOM contains multiple functional groups that play a crucial role in binding with heavy metals. The correlations between DOM properties and heavy metal concentrations are shown in Figure 4b and Figure S7, and Table S6.

Principal component analysis (PCA) was performed using SPSS software to reduce data dimensionality and extract principal components. Significant linear correlations were observed between heavy metals and DOM components, with a statistically valid data structure (Kaiser–Meyer–Olkin (KMO) measure = 0.539, KMO values for all variables > 0.5, Bartlett’ s test *p* < 0.001), confirming the suitability of the data for PCA. Principal component 1 (PC1), explaining 32.2% of the total variance, exhibited high loadings for TOC (0.348), Cu (0.268), Cr (0.251), C2 (0.296), SUVA254 (−0.338), and BIX (−0.295). The high-value points corresponding to PC1 were primarily distributed near traffic-area sampling sites, suggesting that traffic-related emissions (tire wear, lubricants) were the dominant pollution source. Principal component 2 (PC2) accounted for 15.7% of the total variance, with significant loadings for Pb (0.368), Cd (0.303), FI (0.235), TP (−0.397), and TN (−0.351). The high-value points associated with PC2 were mainly clustered near sampling sites in scenic and traffic zones. Principal component 3 (PC3) explained 9.8% of the total variance, dominated by HIX (0.509), Zn (0.450), and C3 (−0.334). The high-value points linked to PC3 were predominantly located in residential and commercial areas. The study revealed that DOM molecular weight is a key factor influencing the migration, toxicity, and bioavailability of heavy metals, with lower DOM molecular weight exerting more pronounced effects on heavy metals [63]. The PCA results effectively elucidated the spatial heterogeneity of pollution sources and the interactions between DOM and heavy metals, providing a scientific basis for targeted pollution control strategies.

In the traffic zone, Cu was significantly and negatively correlated with SUVA254/260, inferring that Cu complexed with DOM fractions, thus reducing bioavailability. Ni was significantly correlated with C1 fractions, which may have been a result of the promotion of their natural transport by C1. Significant correlations between Cu and the C2 and C3 fractions suggest that because of the lower relative molecular mass of these fractions, they are more soluble and therefore may enhance the transport capacity of heavy metals [64]. The highly significant correlation between Cr and V suggests that both may have the same source of contamination. In residential areas, Cu and V showed a positive correlation, indicating they may share common pollution sources. TN and Ni exhibited a positive correlation, suggesting that TN had a stronger cumulative effect on Ni than TP [65]. This could be attributed to nitrogen nutrients carrying higher charges than phosphorus nutrients, making them more prone to electrostatic interactions with heavy metal ions. Cd showed a negative correlation with SUVA254 and SUVA260, implying that Cd forms complexes with low-molecular-weight endogenous DOM components and that the higher aromaticity and humification of DOM weakens its binding capacity with heavy metals. Cu was significantly positively correlated with the C2 component, indicating its involvement in the complexation of xanthate components. In scenic areas, As and Cu displayed a positive correlation, suggesting they may originate from the same pollution source. Ni showed a negative correlation with BIX, indicating that heavy metal content increases with a higher contribution from external sources. Ni also exhibited a negative correlation with SUVA260, implying a relatively low affinity between Ni and DOM during interactions. Zn was significantly positively correlated with the C1 component, possibly because of DOM having a weaker binding capacity for Zn2+ than for other metals. In commercial areas, Ni and Cu showed a negative correlation, suggesting that they derive from different pollution sources. V exhibited a negative correlation with SUVA254 and SUVA260, indicating that V forms complexes with DOM components, thereby reducing the bioavailability of heavy metals. Cd was significantly positively correlated with the C1 and C3 components, likely because DOM contains abundant functional groups that bond with metal ions to form organometallic complexes, influencing the environmental chemical behavior of heavy metal ions [66]. In summary, the characteristics of DOM in RDS are jointly influenced by external pollution and microbial activity, while the content and pollution levels of heavy metals are regulated primarily by anthropogenic factors.

## 4. Conclusions

The pollution levels of heavy metals in Beijing’s RDS followed a descending order: Cd>Pb>Zn>Cu>Cr>Ni>As>V, with transportation zones exhibiting the most severe contamination primarily attributed to anthropogenic activities such as vehicle wear, road paint application, and atmospheric deposition. The $E_{r}^{i}$ analysis indicated that most metals posed low ecological risks across all sampling points, while Cd and Pb showed higher risks at multiple sites, requiring special attention. Health risk assessment revealed that noncarcinogenic risks of heavy metals stemmed primarily from oral ingestion, with children facing significantly higher risks than adults. Moreover, children’s exposure to Cr, Ni, and As exceeded the safety threshold (1 × 10^−4^) for carcinogenic risk, warranting serious concern.

DOM was primarily composed of C1, C2, and C3, with C1 being the most abundant. PCA analysis revealed that traffic emissions were associated with TOC, Cu, Cr, and C2 (PC1, 32.2%), while Pb, Cd, and FI (PC2, 15.7%) reflected characteristics of scenic/traffic areas. In contrast, HIX and Zn (PC3, 9.8%) were linked to residential/commercial zones. DOM in traffic and commercial areas was mainly derived from exogenous sources, whereas residential and scenic areas were dominated by endogenous inputs. DOM regulated heavy metal behavior through complexation and adsorption, with mechanisms varying across functional zones, providing a basis for targeted pollution control. The research findings provide a basis for pollution control in specific regions. Future studies should combine long-term monitoring with molecular-scale analysis to investigate the interaction mechanisms between heavy metals and DOM in RDS under different climatic conditions.

## Data Availability

The raw data of heavy metal concentrations and DOM characteristics in road-deposited sediments are provided in the Supplementary Materials. Processed data for risk assessment and correlation analysis are available from the corresponding author upon reasonable request.

## Supplementary Materials

The following supporting information can be downloaded at: https://www.mdpi.com/article/10.3390/toxics13040308/s1, Figure S1: Average content of heavy metals in different functional areas (a) and ring roads (b); Figure S2: (a) Evaluation results of heavy metal accumulation in different functional areas. Class 1 to class 7 represent unpolluted, unpolluted to moderately polluted, moderately polluted, moderately to heavily polluted, heavily polluted, heavily to extremely polluted, and extremely polluted, respectively. (b) Results of the proportions of potential risks in different functional areas; Figure S3: (a) Traffic, (b) residential, (c) scenic, and (d) commercial three-dimensional fluorescence maps; Figure S4: Contour plots and fluorescence loading of EEM-PARAFAC components; Figure S5: Fluorescence eigenvalues; Figure S6: UV characteristic values; Figure S7: Pearson analysis of heavy metals and DOM fractions in sediments; Table S1: Classification of indices; Table S2: Statistics on heavy metal content in sediments of different functional areas; Table S3: Heavy metal evaluation results; Table S4: Health risk assessment results; Table S5: Optical composition of DOM; Table S6: Principal component extraction results; Formulas S1–S11.
