# Supplementary material for: Risk Assessment of Heavy Metals in Road-Deposited Sediments and Correlation Distribution of DOM and Heavy Metals in Beijing, China
Source: Toxics. 2025 Apr 16;13(4):308. doi: 10.3390/toxics13040308 (PMC12031474; doi:10.3390/toxics13040308)
Supplement: Supplementary file 1 [file toxics-13-00308-s001.zip › toxics-3548092-supplementary.pdf]

Risk Assessment of Heavy Metals in Road-deposited Sediments and Correlation Distribution of DOM and Heavy Metals in Beijing, China

Donghai Yuan[a], Pengmiao Li[a], Chenling Yan[b], Jinggang Wang[c], Xiaochen Bai[a], Yuhang Wei[a], Chen Wang[d], Yingying Kou[a,*]

[a] *Key Laboratory of Urban Stormwater System and Water Environment, Ministry of Education, Beijing University of Civil Engineering and Architecture, Beijing, 100044, China*

[b] *Beijing Key Laboratory of Municipal Solid Waste Detection Analysis and Evaluation, Beijing Municipal Institute of City Management, Beijing 100028, PR China*

[c] *School of Information Technology, Nanchang Vocational University, Nanchang 330007, PR China*

[d] *CAUPD(Beijing) Planning & Design Consultants Co., Ltd., Beijing 100044, PR China*

Supplementary Material

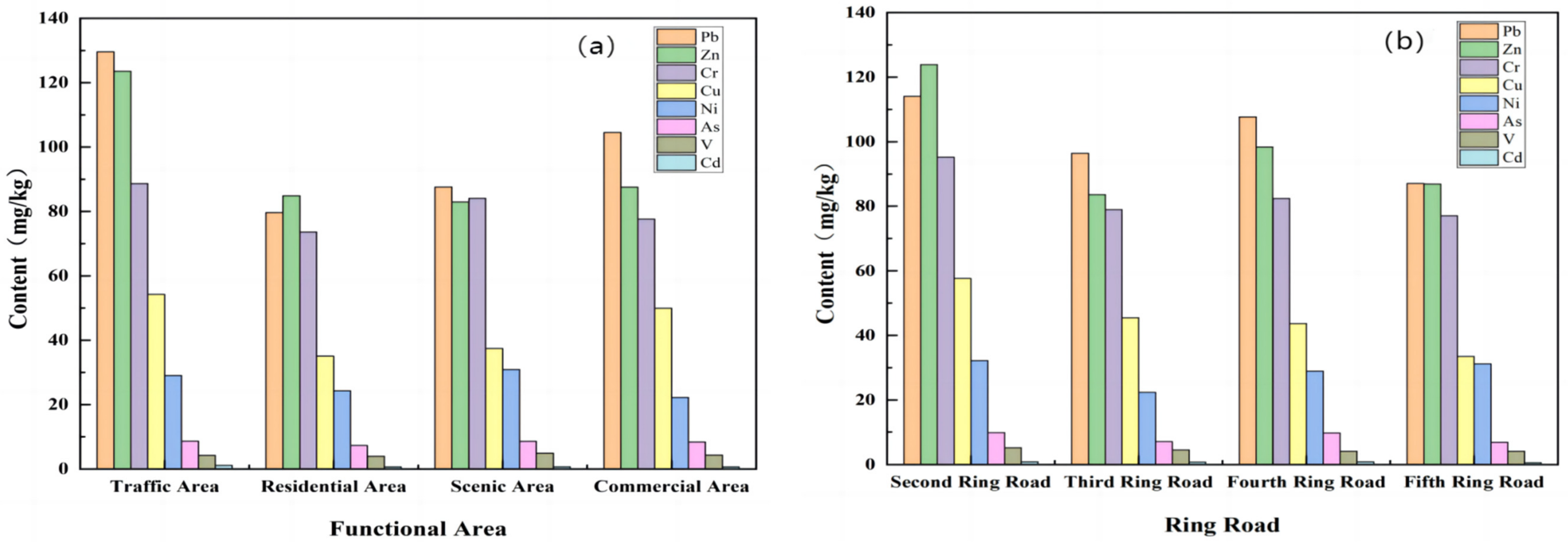

Fig. S1 Average content of heavy metals in different functional areas (a) and ring roads (b).

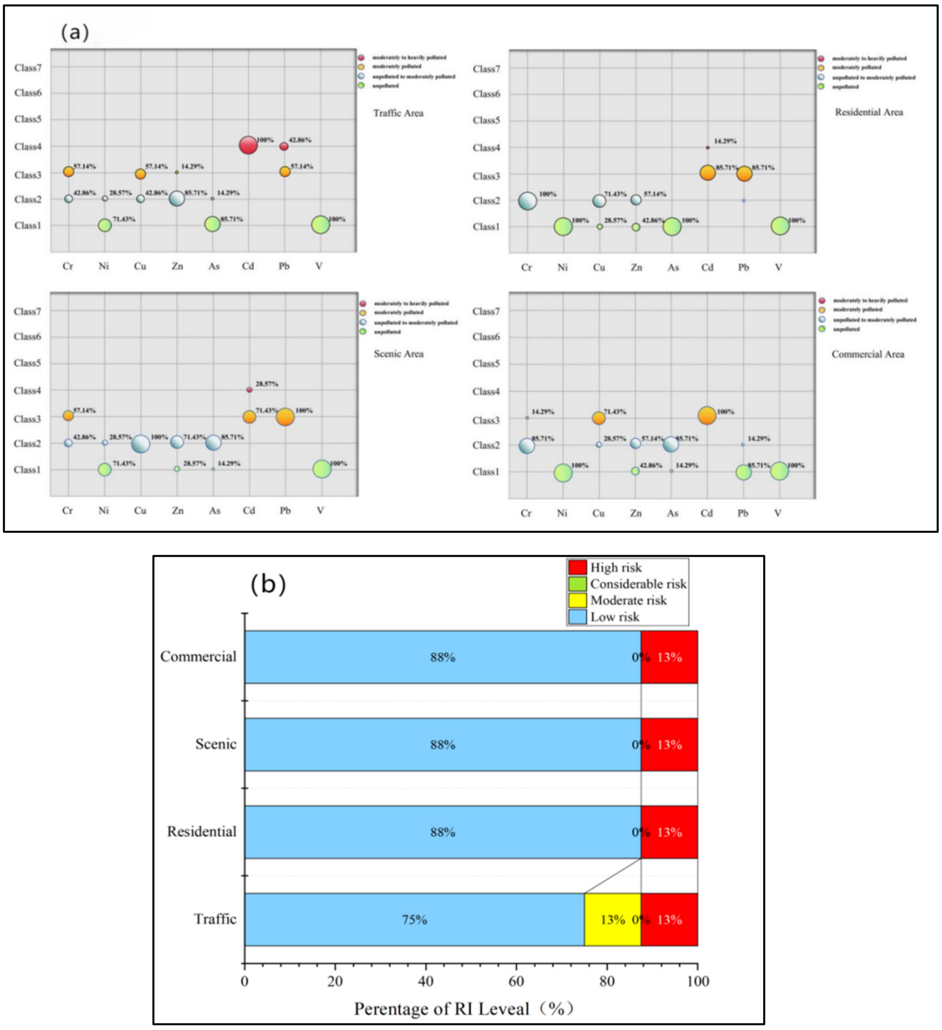

Fig. S2 (a) Evaluation results of heavy metal accumulation in different functional areas. Class 1 to class 7 represent unpolluted, unpolluted to moderately polluted, moderately polluted, moderately to heavily polluted, heavily polluted, heavily to extremely polluted, and extremely polluted, respectively. (b) Results of the proportions of potential risks in different functional areas.

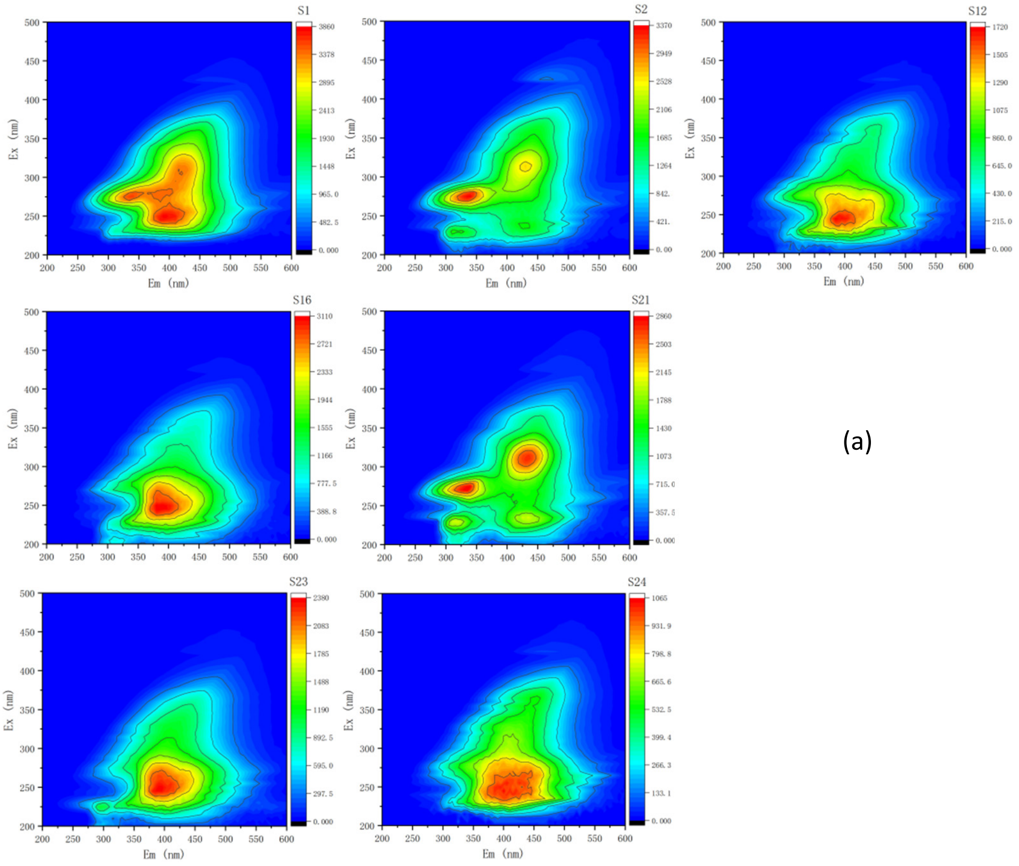
S1
S2
S12
S16
S21
S23
S24
Ex (nm)
Em (nm)
(a)

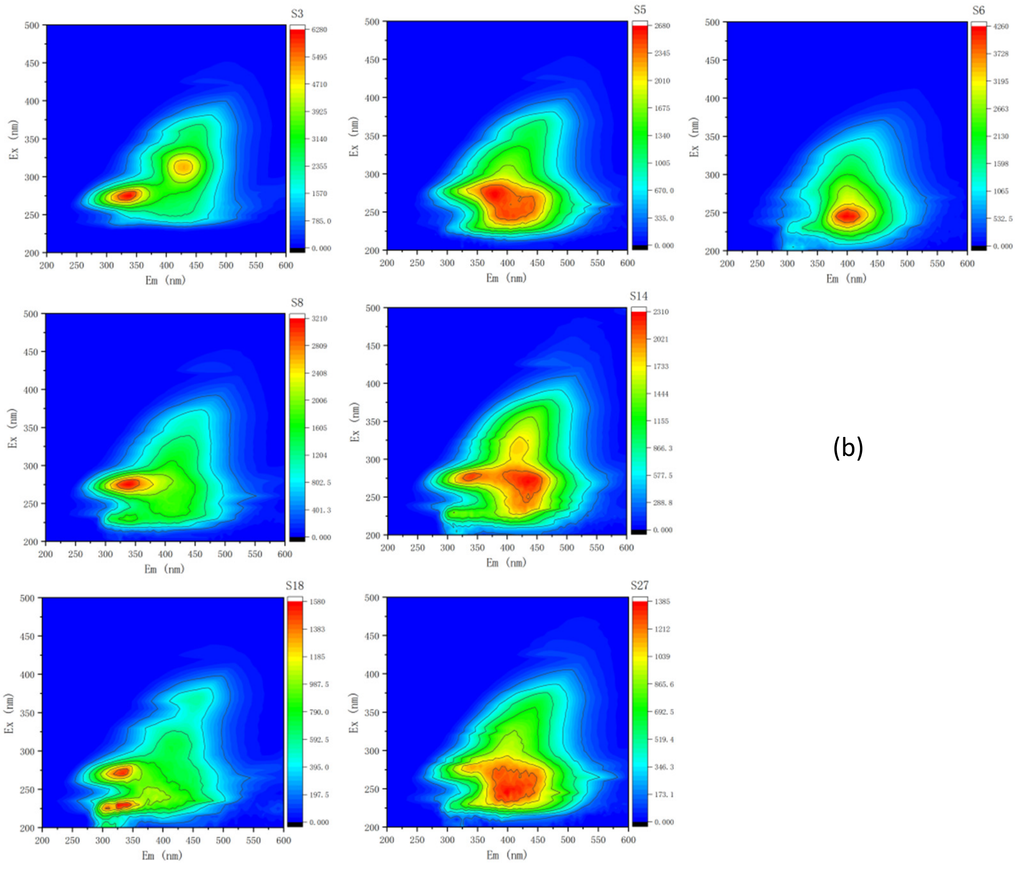
S3
S5
S6
S8
S14
S18
S27
Ex (nm)
Em (nm)
(b)

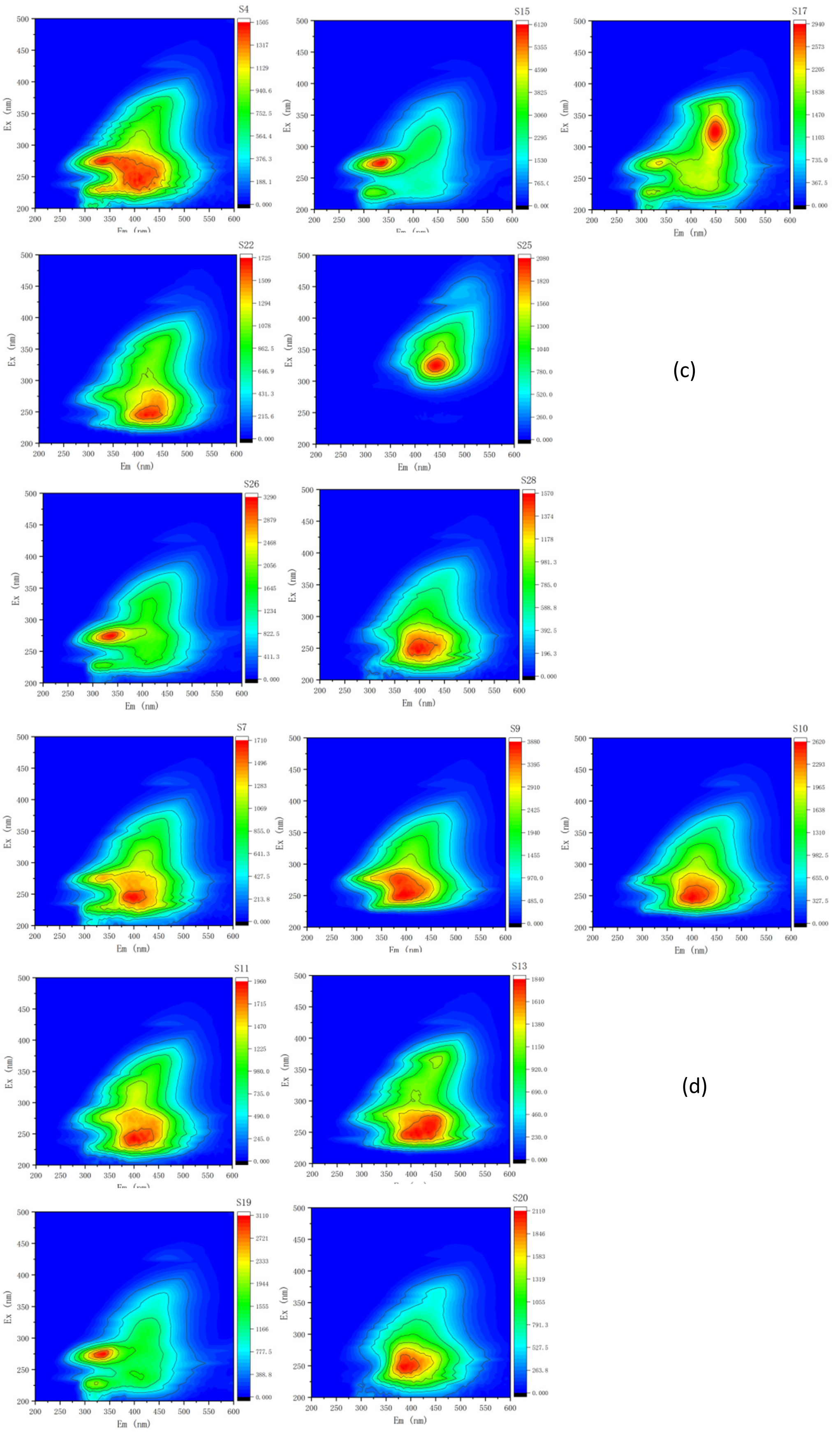

Fig. S3 (a) Traffic, (b) residential, (c) scenic, and (d) commercial three-dimensional fluorescence maps.

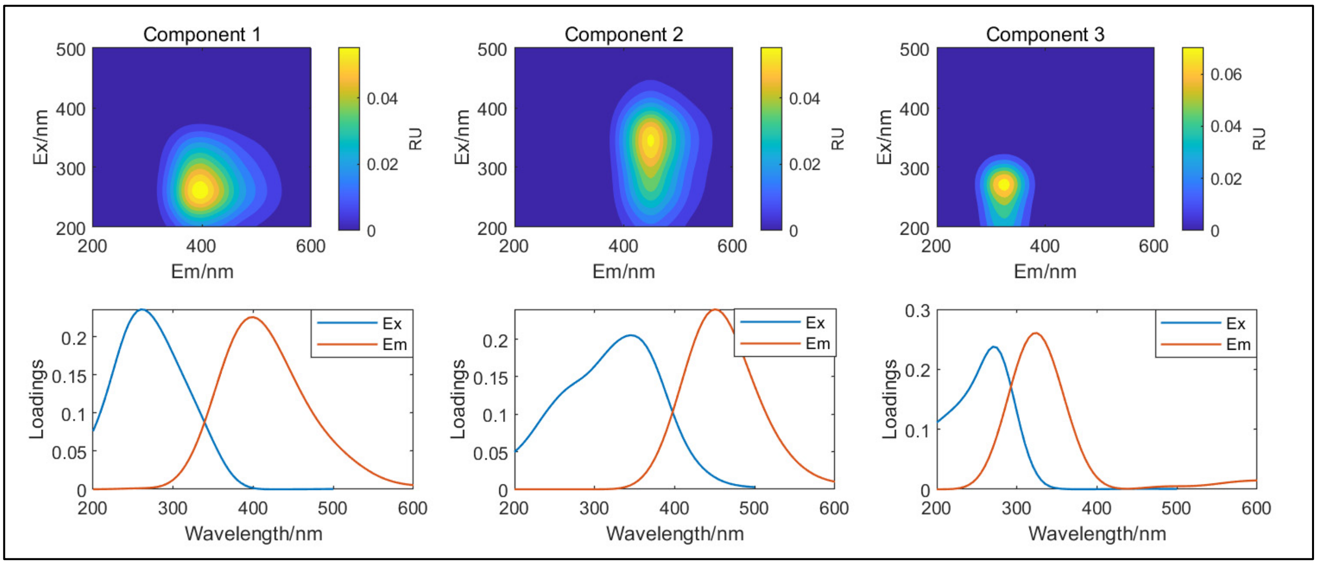

Fig. S4 Contour plots and fluorescence loading of EEM-PARAFAC components.

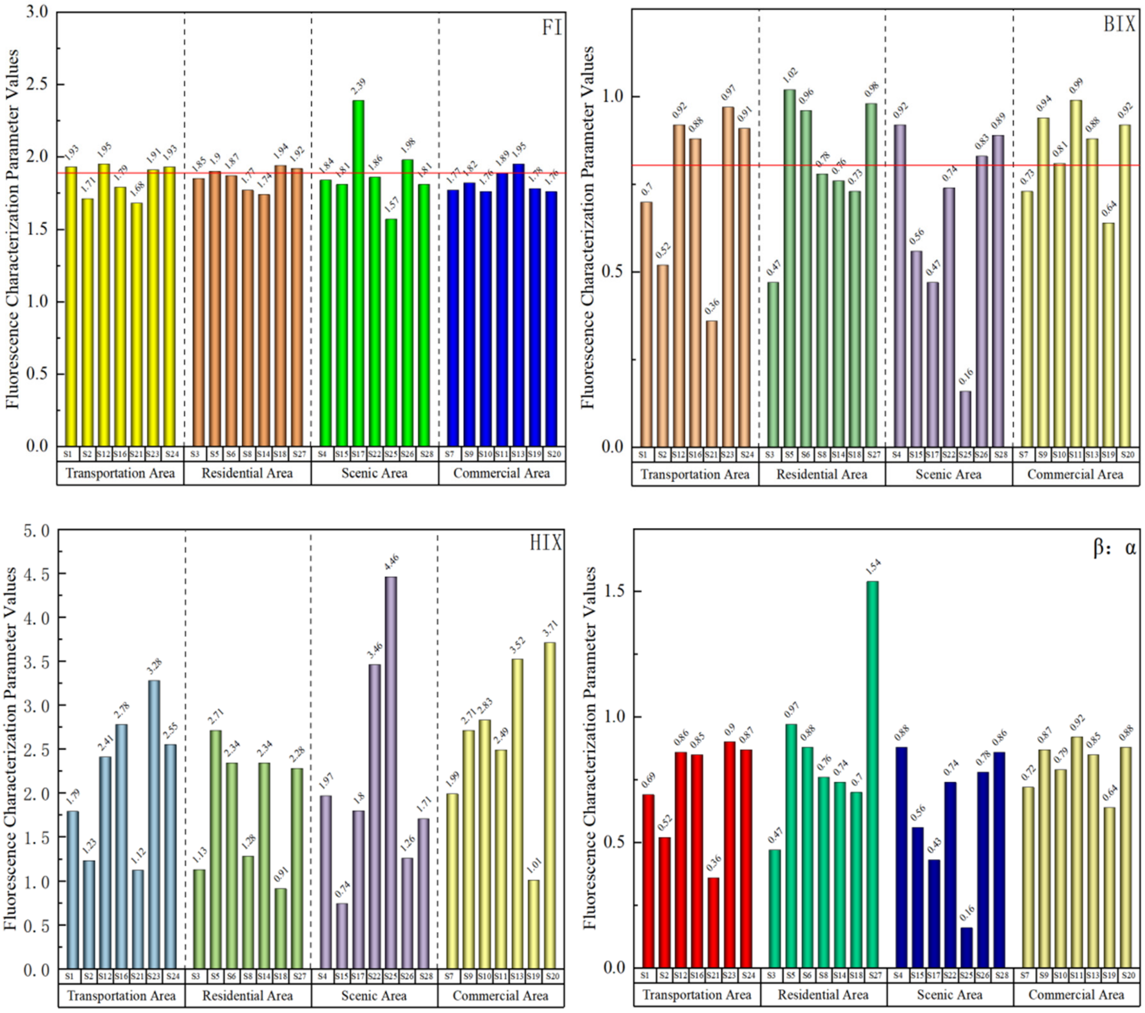

Fig. S5 Fluorescence eigenvalues.

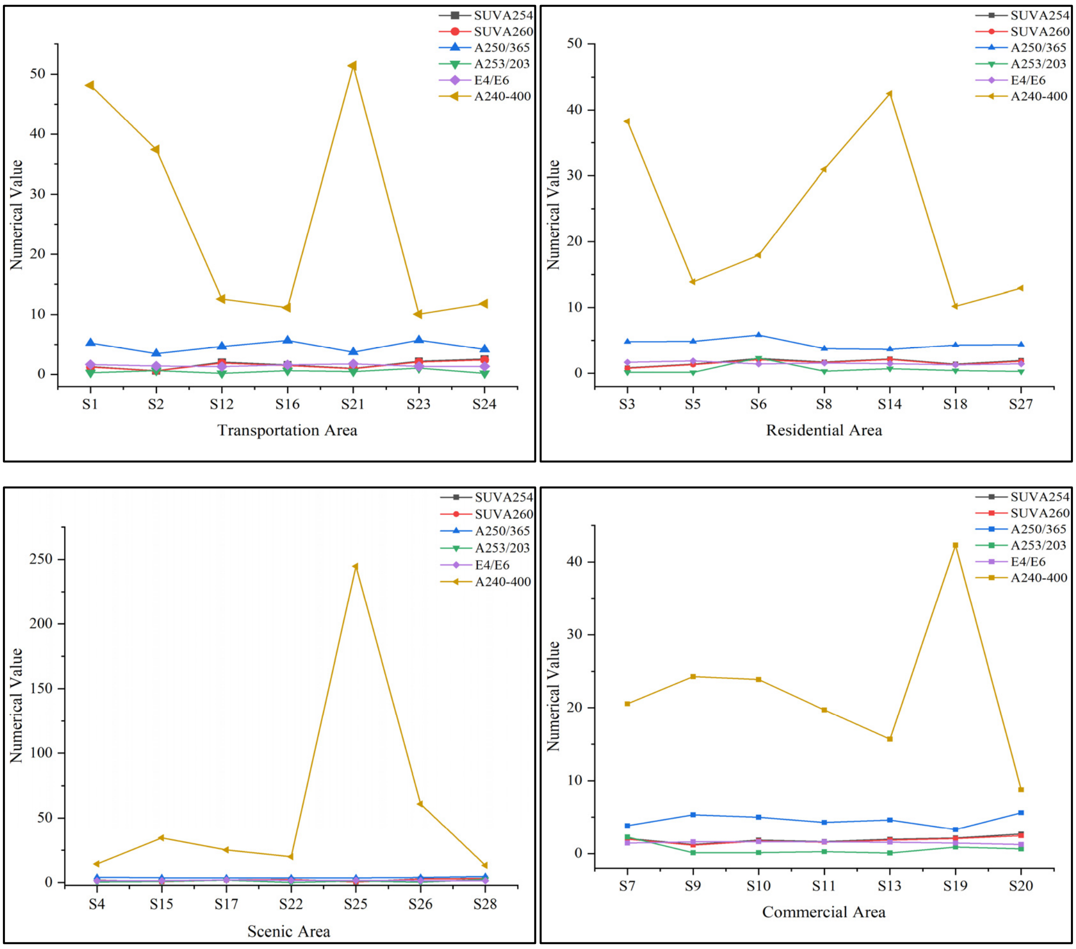

Fig. S6 UV characteristic values.

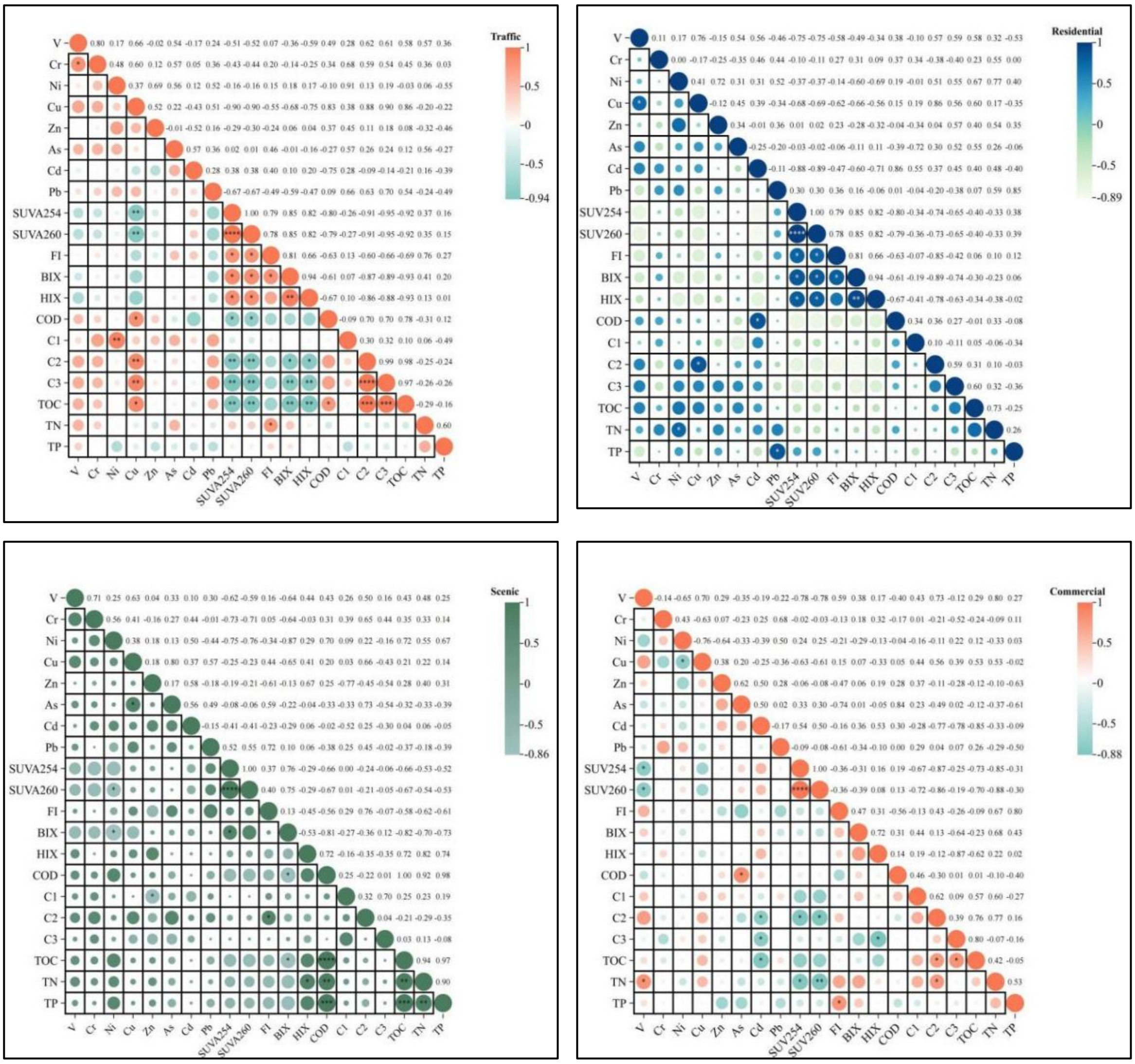

Figure S7. Pearson analysis of heavy metals and DOM fractions in sediments.

Table. S1 Classification of indices.

Methods	Category	Description
Geoaccumulation Index (Igeo)	$I_{geo} \leq 0$	Unpolluted
	$0 < I_{geo} \leq 1$	Unpolluted to moderately polluted
	$1 < I_{geo} \leq 2$	Moderately polluted
	$2 < I_{geo} \leq 3$	Moderately to severely Polluted
	$3 < I_{geo} \leq 4$	Severely polluted
	$4 < I_{geo} \leq 5$	Severely polluted to very severely polluted
	$I_{geo} > 5$	Extremely polluted
Potential Ecological Hazard Index	RI≤150; $E_r^i \leq 40$	Low risk
	150<RI≤300; $40 < E_r^i \leq 80$	Moderate risk
	300<RI≤600; $80 < E_r^i \leq 160$	Considerable risk
	600<RI; $160 < E_r^i \leq 320$	High risk
	$E_r^i > 320$	Extreme risk

Table. S2 Statistics on heavy metal content in sediments of different functional areas.

Heavy metal	Functional area	Maximum value	Minimum value	Mean value	Standard deviation	Coefficient of variation (CV)	Beijing background value [4,45]
Cr	Traffic	124.62	73.78	88.66	15.67	0.18	29.8
	Residential	84.94	50.84	73.60	11.31	0.15	
	Scenic	114.08	45.26	84.05	20.58	0.24	
	Commercial	101.68	65.10	77.59	11.30	0.15	
Ni	Traffic	50.47	21.63	29.01	11.46	0.40	24.7
	Residential	30.90	18.54	24.28	4.19	0.17	
	Scenic	44.29	20.60	30.90	8.04	0.26	
	Commercial	27.81	16.48	22.22	3.77	0.17	
Cu	Traffic	84.15	38.94	54.23	15.87	0.29	18.7
	Residential	45.54	26.40	35.03	6.97	0.20	
	Scenic	44.22	32.34	37.43	4.07	0.11	
	Commercial	64.68	38.61	49.92	8.32	0.17	
Zn	Traffic	184.43	87.72	123.51	33.33	0.27	57.5
	Residential	125.07	28.55	84.86	29.34	0.35	
	Scenic	113.63	59.15	82.94	17.15	0.21	
	Commercial	152.15	32.90	87.54	39.03	0.45	
As	Traffic	14.45	7.34	8.62	2.19	0.25	7.7
	Residential	10.88	1.72	7.29	3.25	0.45	
	Scenic	11.77	6.38	8.59	2.00	0.23	
	Commercial	12.53	3.30	8.35	2.78	0.33	
Cd	Traffic	1.29	0.86	1.11	0.14	0.13	0.12
	Residential	0.77	0.55	0.64	0.07	0.10	

	Scenic	0.81	0.58	0.67	0.08	0.12	
	Commercial	0.68	0.63	0.65	0.02	0.03	
Pb	Traffic	171.00	90.00	129.60	27.72	0.21	24.6
	Residential	90.90	72.90	79.65	5.53	0.07	
	Scenic	99.90	76.50	87.56	9.09	0.10	
	Commercial	175.50	75.60	104.53	31.23	0.30	
V	Traffic	5.83	3.44	4.20	0.90	0.21	79.2
	Residential	4.79	2.72	3.96	0.76	0.19	
	Scenic	5.79	2.96	4.91	0.87	0.18	
	Commercial	5.14	3.49	4.32	0.58	0.13	

Table. S3 Heavy metal evaluation results,

Heavy metal	Functional area	Igeo	Contamination level	RI	E_r^i	Ecological Risks
Cr	Traffic	0.72~1.48	Unpolluted to moderately polluted	44.07	6.30	Minor
	Residential	0.19~0.93	Unpolluted to moderately polluted	34.58	4.94	Minor
	Scenic	0.02~1.35	Unpolluted to moderately polluted	39.49	5.64	Minor
	Commercial	0.54~1.19	Unpolluted to moderately polluted	36.45	5.21	Minor
Ni	Traffic	-0.78~0.45	Unpolluted to moderately polluted	45.45	6.49	Minor
	Residential	-1~-0.26	Uncontaminated	34.40	4.91	Minor
	Scenic	-0.85~0.26	Unpolluted to moderately polluted	43.79	6.26	Minor
	Commercial	-1.17~-0.41	Uncontaminated	31.48	4.50	Minor
Cu	Traffic	0.47~1.58	Unpolluted to moderately polluted	106.76	15.25	Minor
	Residential	-0.09~0.70	Unpolluted to moderately polluted	65.56	9.37	Minor
	Scenic	0.21~0.66	Unpolluted to moderately polluted	70.06	10.01	Minor
	Commercial	0.46~1.21	Unpolluted to moderately polluted	93.44	13.35	Minor
Zn	Traffic	0.02~1.10	Unpolluted to moderately polluted	15.24	2.18	Minor
	Residential	-1.60~0.54	Unpolluted to moderately polluted	10.33	1.48	Minor
	Scenic	-0.54~0.40	Unpolluted to moderately polluted	10.10	1.44	Minor
	Commercial	-1.39~0.82	Unpolluted to moderately polluted	10.66	1.52	Minor
As	Traffic	-0.65~0.32	Unpolluted to moderately polluted	173.97	24.85	Minor
	Residential	-2.75~-0.09	Uncontaminated	107.98	15.43	Minor
	Scenic	-0.86~0.03	Unpolluted to moderately polluted	95.67	13.67	Minor
	Commercial	-1.81~0.12	Unpolluted to moderately polluted	122.41	17.49	Minor
Cd	Traffic	2.25~2.84	Moderately to severely polluted	1987.20	283.89	Strong
	Residential	1.61~2.09	Moderately to severely polluted	1128.00	161.14	Strong

	Scenic	1.68~2.16	Moderately to severely polluted	1174.40	167.77	Strong
	Commercial	1.80~1.91	Moderately to severely polluted	1144.00	163.43	Strong
Pb	Traffic	1.29~2.21	Moderately to severely polluted	191.89	27.41	Medium
	Residential	0.98~1.30	Moderately polluted	113.32	16.19	Minor
	Scenic	1.05~1.44	Moderately polluted	124.57	17.80	Minor
	Commercial	1.03~2.25	Moderately polluted	148.72	21.25	Minor
V	Traffic	-5.11~-4.35	Uncontaminated	0.78	0.11	Minor
	Residential	-5.45~4.63	Uncontaminated	0.70	0.10	Minor
	Scenic	-5.33~4.36	Uncontaminated	0.87	0.12	Minor
	Commercial	-5.09~-4.53	Uncontaminated	0.76	0.11	Minor

Table. S4 Health risk assessment results.

		Noncarcinogenic risks								Carcinogenic risks							
		HQ_{ing}		HQ_{inh}		HQ_{dermal}		HI		CR_{ing}		CR_{inh}		CR_{dermal}		CR	
		**Children**	**Adults**	**Children**	**Adults**	**Children**	**Adults**	**Children**	**Adults**	**Children**	**Adults**	**Children**	**Adults**	**Children**	**Adults**	**Children**	**Adults**
Traffic	Cr	2.40E-01	9.39E-03	8.85E-04	1.95E-04	1.34E-02	7.49E-04	2.54E-01	1.03E-02	6.01E-04	2.35E-05	1.41E-06	2.35E-07	6.72E-05	3.75E-06	6.70E-04	2.75E-05
	Ni	2.05E-02	8.03E-04	1.17E-03	9.28E-08	1.15E-03	6.40E-05	2.28E-02	8.67E-04	3.44E-04	1.35E-05	9.63E-09	1.61E-09	9.65E-07	5.38E-08	3.45E-04	1.36E-05
	Cu	1.82E-02	7.14E-04	5.56E-07	8.50E-08	1.70E-04	9.49E-06	1.84E-02	7.24E-04	-	-	-	-	-	-	-	-
	Zn	5.33E-03	2.09E-04	5.09E-07	2.49E-08	7.47E-05	4.16E-06	5.41E-03	2.13E-04	-	-	-	-	-	-	-	-
	As	4.03E-01	1.58E-02	1.49E-07	4.58E-06	3.93E-03	2.19E-04	4.07E-01	1.60E-02	1.81E-04	7.09E-06	5.10E-08	8.51E-09	5.11E-06	2.85E-07	1.86E-04	7.38E-06
	Cd	1.45E-02	5.68E-04	2.74E-05	6.77E-08	8.13E-04	4.53E-05	1.53E-02	6.13E-04	9.15E-05	3.58E-06	2.56E-09	4.26E-10	2.56E-07	1.43E-08	9.18E-05	3.59E-06
	Pb	4.92E-01	1.93E-02	4.06E-07	2.28E-06	9.20E-03	5.13E-04	5.01E-01	1.98E-02	1.47E-05	5.74E-07	2.02E-09	3.38E-10	8.21E-08	4.58E-09	1.48E-05	5.79E-07
	V	4.05E-03	1.59E-04	1.37E-05	1.48E-04	4.36E-04	2.43E-05	4.50E-03	3.31E-04	-	-	-	-	-	-	-	-
Residential	Cr	1.88E-01	7.37E-03	4.21E-07	1.53E-04	8.69E-04	5.88E-04	1.64E-02	8.11E-03	4.71E-04	1.85E-05	1.10E-06	1.84E-07	5.27E-05	2.94E-06	5.25E-04	2.16E-05
	Ni	1.55E-02	6.07E-04	3.13E-07	7.03E-08	1.04E-04	4.85E-05	1.13E-02	6.56E-04	2.61E-04	1.02E-05	7.29E-09	1.22E-09	7.30E-07	4.07E-08	2.62E-04	1.02E-05
	Cu	1.12E-02	4.38E-04	1.01E-07	5.22E-08	5.06E-05	5.83E-06	3.67E-03	4.44E-04	-	-	-	-	-	-	-	-
	Zn	3.62E-03	1.42E-04	2.12E-05	1.69E-08	3.03E-03	2.82E-06	3.14E-01	1.45E-04	-	-	-	-	-	-	-	-
	As	3.11E-01	1.22E-02	2.30E-07	3.53E-06	4.62E-04	1.69E-04	8.70E-03	1.24E-02	1.40E-04	5.47E-06	3.93E-08	6.56E-09	3.94E-06	2.20E-07	1.44E-04	5.70E-06

	Cd	8.24E-03	3.23E-04	8.08E-06	3.84E-08	5.43E-03	2.57E-05	2.96E-01	3.49E-04	5.19E-05	2.03E-06	1.45E-09	2.42E-10	1.45E-07	8.11E-09	5.20E-05	2.04E-06
	Pb	2.91E-01	1.14E-02	7.91E-04	1.35E-06	3.90E-04	3.03E-04	4.80E-03	1.17E-02	8.66E-06	3.39E-07	1.20E-09	1.99E-10	4.85E-08	2.70E-09	8.71E-06	3.42E-07
	V	3.62E-03	1.42E-04	7.91E-04	1.32E-04	3.90E-04	2.17E-05	4.80E-03	2.96E-04	-	-	-	-	-	-	-	-
	Cr	2.15E-01	8.41E-03	1.05E-03	1.75E-04	1.20E-02	6.71E-04	2.28E-01	9.26E-03	5.38E-04	2.11E-05	1.26E-06	2.10E-07	6.02E-05	3.36E-06	5.99E-04	2.47E-05
	Ni	1.98E-02	7.73E-04	5.36E-07	8.94E-08	1.11E-03	6.17E-05	2.09E-02	8.35E-04	3.32E-04	1.30E-05	9.27E-09	1.55E-09	9.29E-07	5.18E-08	3.33E-04	1.31E-05
	Cu	1.20E-02	4.68E-04	3.34E-07	5.58E-08	1.12E-04	6.23E-06	1.21E-02	4.74E-04	-	-	-	-	-	-	-	-
Scenic	Zn	3.53E-03	1.38E-04	9.88E-08	1.65E-08	4.95E-05	2.76E-06	3.58E-03	1.41E-04	-	-	-	-	-	-	-	-
	As	3.66E-01	1.43E-02	2.49E-05	4.16E-06	3.57E-03	1.99E-04	3.70E-01	1.45E-02	1.65E-04	6.45E-06	4.63E-08	7.73E-09	4.64E-06	2.59E-07	1.70E-04	6.72E-06
	Cd	8.58E-03	3.36E-04	2.40E-07	4.00E-08	4.80E-04	2.68E-05	9.06E-03	3.63E-04	5.41E-05	2.12E-06	1.51E-09	2.52E-10	1.51E-07	8.44E-09	5.43E-05	2.13E-06
	Pb	3.20E-01	1.25E-02	8.89E-06	1.48E-06	5.97E-03	3.33E-04	3.26E-01	1.28E-02	9.52E-06	3.72E-07	1.31E-09	2.19E-10	5.33E-08	2.97E-09	9.57E-06	3.75E-07
	V	4.48E-03	1.75E-04	9.80E-04	1.64E-04	4.83E-04	2.69E-05	5.94E-03	3.66E-04	-	-	-	-	-	-	-	-
	Cr	1.98E-01	7.77E-03	9.69E-04	1.62E-04	1.11E-02	6.20E-04	2.10E-01	8.55E-03	4.97E-04	1.95E-05	1.16E-06	1.94E-07	5.56E-05	3.10E-06	5.54E-04	2.28E-05
	Ni	1.42E-02	5.56E-04	3.85E-07	6.43E-08	7.95E-04	4.44E-05	1.50E-02	6.00E-04	2.39E-04	9.34E-06	6.67E-09	1.11E-09	6.68E-07	3.73E-08	2.40E-04	9.38E-06
Comm	Cu	1.60E-02	6.25E-04	4.46E-07	7.44E-08	1.49E-04	8.31E-06	1.61E-02	6.33E-04	-	-	-	-	-	-	-	-
ercial	Zn	3.73E-03	1.46E-04	1.04E-07	1.74E-08	5.22E-05	2.91E-06	3.78E-03	1.49E-04	-	-	-	-	-	-	-	-
	As	3.56E-01	1.39E-02	2.43E-05	4.05E-06	3.48E-03	1.94E-04	3.60E-01	1.41E-02	1.60E-04	6.27E-06	4.51E-08	7.52E-09	4.51E-06	2.52E-07	1.65E-04	6.53E-06
	Cd	8.36E-03	3.27E-04	2.34E-07	3.90E-08	4.68E-04	2.61E-05	8.83E-03	3.53E-04	5.27E-05	2.06E-06	1.47E-09	2.45E-10	1.47E-07	8.22E-09	5.28E-05	2.07E-06

Pb	3.82E-01	1.49E-02	1.06E-05	1.77E-06	7.13E-03	3.98E-04	3.89E-01	1.53E-02	1.14E-05	4.45E-07	1.57E-09	2.62E-10	6.36E-08	3.55E-09	1.15E-05	4.49E-07
V	3.95E-03	1.55E-04	8.63E-04	1.44E-04	4.25E-04	2.37E-05	5.24E-03	3.23E-04	-	-	-	-	-	-	-	-

Table. S5 Optical composition of DOM.

Functional Area		SUVA254	SUVA260	A250/365	A253/203	E4/E6	A240-400	FI	BIX	HIX	β：α	C1	C2	C3
Transportation Area	Maximum value	2.59	2.43	5.74	1.06	1.78	51.38	1.95	0.97	3.28	0.9	1.80	0.85	1.73
	Minimum value	0.62	0.59	3.48	0.2	1.32	10.08	1.68	0.36	1.12	0.36	0.52	0.21	0.26
	Mean value	1.63	1.53	4.68	0.5	1.5	26.09	1.84	0.75	2.17	0.72	1.05	0.50	0.97
	Coefficient of variation	262.90%	257.48%	134.56%	252.14%	113.74%	258.80%	6.19%	31.08%	37.40%	28.96%	0.43	0.57	0.66
Residential Area	Maximum value	2.26	2.12	5.84	2.35	1.9	42.5	1.94	1.02	2.71	1.54	1.83	0.70	0.95
	Minimum value	0.84	0.77	3.66	0.15	1.3	10.21	1.74	0.47	0.91	0.47	0.47	0.20	0.53
	Mean value	1.68	1.59	4.52	0.63	1.55	23.81	1.86	0.81	1.86	0.87	1.07	0.39	0.72
	Coefficient of variation	30.19%	30.35%	16.49%	123.67%	12.84%	55.45%	4.07%	23.56%	38.93%	38.79%	0.41	0.43	0.23
Scenic Area	Maximum value	3.27	3.03	4.74	2.57	2.07	244.84	2.39	0.92	4.46	0.88	1.01	1.00	1.56
	Minimum value	0.56	0.54	3.63	0.12	1.44	13.31	1.57	0.16	0.74	0.16	0.68	0.22	0.25
	Mean value	1.99	1.89	3.93	1.09	1.65	59.01	1.89	0.65	2.2	0.63	0.82	0.52	0.69
	Coefficient of variation	51.82%	51.03%	10.16%	81.53%	12.95%	141.58%	13.23%	42.02%	59.16%	41.79%	0.13	0.52	0.65
	Maximum	2.69	2.45	5.61	2.27	1.63	42.3	1.95	0.99	3.71	0.92	1.81	0.66	1.71

**Commercial Area**	value													
	Minimum value	1.25	1.16	3.29	0.07	1.24	8.77	1.76	0.64	1.01	0.64	0.79	0.21	0.25
	Mean value	1.94	1.82	4.56	0.63	1.5	22.17	1.82	0.84	2.61	0.81	1.05	0.43	0.75
	Coefficient of variation	22.92%	22.41%	18.15%	125.52 %	9.22%	46.71%	4.08%	14.78%	35.19%	12.30%	0.35	0.32	0.66

Table. S6 Principal component extraction results.

	PC1	PC2	PC3
V	0.24601	-0.03212	0.00995
Cr	0.25078	0.11456	0.13884
Ni	0.20742	0.03111	0.26001
Cu	0.26835	0.21911	0.07415
Zn	0.10015	0.13371	0.45035
As	0.06397	0.26578	0.13443
Cd	0.16578	0.30282	0.2563
Pb	0.16514	0.36772	0.18857
SUVA254	-0.33778	0.20409	0.01227
SUVA260	-0.3349	0.20618	0.0088

FI	-0.12777	0.23517	-0.11169
BIX	-0.29492	0.13225	0.16121
HIX	-0.12357	-0.14507	0.50852
COD	0.23455	-0.34731	0.10124
C1	0.12357	0.05013	0.16291
C2	0.29571	0.1568	-0.24639
C3	0.24526	0.11708	-0.33375
TOC	0.34843	-0.02982	-0.13188
TN	0.09181	-0.35106	0.24198
TP	-0.01139	-0.39732	0.05714

Formula

Geoaccumulation index

The geoaccumulation index is the most commonly used index to determine the level of contamination of one element in soils. It is expressed as follows [28-30]:

$$I_{geo} = \log_2\left[\frac{C_i}{k\times B_i}\right]$$

(S1)

where C_i represents the concentration of elemental content within sediment ($mg\cdot kg^{-1}$); B_i is the geochemical background value for metal n ($mg\cdot kg^{-1}$); and k is the correction factor, which is generally taken as 1.5, as ascertained by the computation of the Igeo and pollution level as shown in Table. S1.

Potential ecological risk index

Hakanson is a Swedish scientist who proposed the method of potential ecological risk index (PERI) [31]. PERI quantitatively expresses the potential ecological risk of a given pollutant and is widely used in multielement pollution assessment. It is expressed as follows [32-35]:

$$C_f^i = C_s^i / C_n^i$$

(S2)

$$E_r^i = T_r^i \times C_f^i$$

(S3)

$$\mathrm{RI} = \sum_{i=1}^{n} \mathrm{E_r^i} = \sum_{i=1}^{n} \mathrm{T_r^i} \times \mathrm{C_f^i} = \sum_{i=1}^{n} \mathrm{T_r^i} \times \frac{\mathrm{C_s^i}}{\mathrm{C_n^i}}$$

(S4)

where RI expresses the combined multifactor environmental risk indicator; the environmental hazard indicator for heavy metals of category i is represented by E_r^i; and C_f^i is the contamination factor, C_s^i represents measured data, and C_n^i is the geochemical background value for the element. T_r^i represents the toxicity response factor for heavy metals, with coefficients of Cu=Pb=Ni=5, As=10, Cd=30, V=Cr=2, and Zn=1. E_r^i, the risk indicator (RI) for each element, can be classified into five levels. The multifactor environmental risk indicator RI is the combined value of potential coefficients of several pollutants at the sampling site and can be classified into four levels, as shown in Table S1.

Human Health Risk Assessment Method

To evaluate the danger to human health as a result of exposure to the metals in RDS, chronic daily intake (CDI) was utilized [36,37].

$$CDI_{ing} = \frac{CS \times EF \times ED \times IRS}{BW \times AT \times 10^6} \quad (S5)$$

$$CDI_{inh} = \frac{PM \times CS \times ET \times EF \times IR_{air} \times ED}{BW \times PEF \times AT}$$

(S6)

$$CDI_{dermal} = \frac{CS \times SA \times AF \times EF \times ED \times ABS}{BW \times AT \times 10^6} \quad (S7)$$

where CDI_{ing}, CDI_{inh} and CDI_{dermal} are the average daily intakes through direct ingestion, inhalation, and dermal contact absorption, respectively, $mg \cdot kg^{-1} \cdot d^{-1}$; CS denotes the content of heavy metal elements in RDS, $mg \cdot kg^{-1}$; PM refers to the ambient concentration of particulate matter in the target area (0.146 $mg \cdot kg^{-1}$); IRS represents the ingestion rate of 100 $mg \cdot d^{-1}$ according to the guidelines of [38]; EF is the exposure frequency, 365 $d \cdot a^{-1}$; ED is exposure duration; BW is body weight, kg: with adults being 70 kg and children being 15 kg; AT is the average exposure time, d; IR_{air} denotes the inhalation rate of air (20 $m^3 \cdot d^{-1}$); PEF is the particulate emission factor, $m^3 \cdot kg^{-1}$ [39]; SA is the skin-

exposed area, cm^2, being 5700 $cm^2 \cdot d^{-1}$ for adults and 2800 $cm^2 \cdot d^{-1}$ for children; and AF is the skin adherence factor, $mg \cdot cm^{-2}$, being 0.07 $mg \cdot cm^{-2}$ for adults and 0.2 $mg \cdot cm^{-2}$ for children [38], with a conversion factor of 10^6 to convert from kg to mg.

The following equations were used to assess the hazard quotient (HQ) and hazard index (HI) [40]:

$$HQ = \frac{CDI_i}{R_f D} \quad \text{(S8)}$$

$$HI = \sum_{i=1}^{n} HQ_k = HQ_{inh} + HQ_{ing} + HQ_{dermal}$$

(S9)

where $R_f D$ is the reference dose, $mg \cdot kg^{-1} \cdot d^{-1}$, and HI is the hazard index. For HI, if HI≤1, the total noncarcinogenic risk of multiple heavy metals to humans is negligible; if HI>1, the total noncarcinogenic risk of multiple heavy metals to humans is unacceptable.

The formula for assessing carcinogenic risk (CR) was as follows:

(S10) $$CR_i = CSF_i \times CDI_i$$

(S11) $$CR = \sum_{i=1}^{n} CR_i$$

where CSF_i denotes the carcinogenicity slope factor. For CR, if $CR_i < 10^{-6}$, the carcinogenic risk to humans from multiple heavy metals is negligible; if $CR_i > 10^{-4}$, the total carcinogenic risk to humans from multiple heavy metals is unacceptable [41,42].

Reference

4. Men, C., Liu, R.M., Xu, F., Wang, Q.R., Guo, L.J., Shen, Z.Y. Pollution characteristics, risk assessment, and source apportionment of heavy metals in road dust in Beijing, China. Sci. Total Environ, 2018. 612, 138-147.

28. Zahra, A., Hashmi, M.Z., Malik, R.N., Ahmed, Z. Enrichment and geo-accumulation of heavy metals and risk assessment of sediments of the Kurang Nallah—Feeding tributary of the Rawal Lake Reservoir, Pakistan. Sci. Total Environ, 2014. 470-471, 925-933.

29. Pathak, A.K., Kumar, R., Kumar, P., Yadav, S. Sources apportionment and spatio-temporal changes in metal pollution in surface and sub-surface soils of a mixed type industrial area in India. J. Geochem. Explor, 2015. 159, 169-177.

30. Li, J. , Zuo, Q. , Feng, F. , Jia, H. , & Ji, Y. Pollution characteristics, bioavailability, and risk assessment of heavy metals in urban road dust from zhengzhou, china. Environ Geochem Hlth, 2024. 46(11), 1-19.

31. Hakanson, L. An Ecological Risk Index for Aquatic Pollution Control. a sedimentological Approach. Water Res, 1980. 975-1001.

32. Kusin, F.M., Rahman, M.S.A., Madzin, Z., Jusop, S., Mohamat-Yusuff, F., Ariffin, M., et al. The occurrence and potential ecological risk assessment of bauxite mine-impacted water and sediments in Kuantan, Pahang,Malaysia. Environ. Sci. Pollut. Res 2016. 24, 1306-1321.

33. Ayari, J., Barbieri, M., Agnan, Y., Sellami, A., Braham, A., Dhaha, F., et al. Trace element contamination in the mine-affected stream sediments of Oued Rarai in north-western Tunisia: a river basin scale assessment. Environ. Geochem. Health, 2021. 43, 4027-4042.

34. Tsogtbaatar, U. , Huo, L. , Jiao, L. , Dalantai, S. , An, Y. , & Batsaikhan, B. , et al. Ecological risk evaluation of heavy metals based on hyperspectral: a case study of rice paddy soil in xiangtan county, china. Environ Monit Assess, 2025. 197(4), 1-13.

35. Vasudhevan, P., Pu, S., Ayyamperumal, R., etal. Pollution assessment, ecological risk and source identifcation of heavy metals in paddy soils and rice grains from Salem, South India. J Hazard Mater Adv, 2025. 17, 100526.

36. Bhuyan, M. S., Haider, S. M. B., Meraj, G., Bakar, M. A., Islam, M. T., Kunda, M., & Bhat, M.

A. Assessment of heavy metal contamination in beach sediments of eastern St. Martin's island, Bangladesh: implications for environmental and human health risks. Water, 2023. 15 (13), 2494.

37. Gao, Z., Song, J., Abeysekara, S. D., Liu, J., Zhang, Y., Li, Q., Jiang, B. Soil Heavy Metal Pollution in Upstream Bailang River, Eastern China: Spatial Analysis, Health Risks, and Pollution Source Identification. Soil Sediment Contam, 2024. 1–27.

38. United States Environmental Protection Agency (USEPA). (2022). Exposure Factors Handbook 2011 Edition (Final).

39. United States Environmental Protection Agency (USEPA).. Supplemental Guidance for Developing Soil Screening Levels for Superfund Sites OSWER, 2002. 9355.4-24.

40. United States Environmental Protection Agency (USEPA). (2000). Risk-Based Concentration Table; USEPA: Washington, DC, USA.

41. Kalipci, E., Cüce, H., Ustao glu, F., Dereli, M.A., Türkmen, M. Toxicological health risk analysis of hazardous trace elements accumulation in the edible fish species of the Black Sea in Türkiye using multivariate statistical and spatial assessment. Environ.Toxicol. Pharmacol, 2023. 97, 104028.

42. IARC. Cancer, Agents Classified by the IARC Monographs. (2011). IARC Monographs on the Evaluation of Carcinogenic Risks to Humans; IARC: Geneva, Switzerland.

45. Li, R., Cai, G., Wang, J., Ouyang, W., Cheng, H., Lin, C. Contents and chemical forms of heavy metals in school and roadside topsoils and road-surface dust of Beijing. J. Soils Sediments, 2014. 14, 1806-1817.
